# Etiology and Antimicrobial Resistance Patterns of Acute Bacterial Meningitis in Children: A 10-Year Referral Hospital-Based Study in Northwest Iran

**DOI:** 10.5812/ircmj.17616

**Published:** 2014-07-05

**Authors:** Babak Abdinia, Mohammad Ahangarzadeh Rezaee, Shahram Abdoli Oskouie

**Affiliations:** 1 Department of Pediatrics, Faculty of Medicine, Tabriz University of Medical Sciences, Tabriz, IR Iran; 2 Tabriz Infectious and Tropical Diseases Research Center, Tabriz University of Medical Sciences, Tabriz, IR Iran

**Keywords:** Bacterial Meningitis, Child, Antibiotics

## Abstract

**Background:**

Bacterial meningitis is still considered as one of the most dangerous infectious diseases, which causes numerous complications and high mortality if not diagnosed and treated timely.

**Objectives:**

This study was performed to determine antimicrobial resistance patterns of bacterial pathogens isolated from acute bacterial meningitis at Tabriz Children Educational-Health Care Center in Iran.

**Patients and Methods:**

In a retrospective study (from 2003 through 2013), all patients with bacterial meningitis were identified by cerebrospinal fluids with positive results in culture (107 cases). Patients' necessary data was recorded in a questionnaire. Furthermore, the results of simultaneous blood culture were also examined. Ultimately, antimicrobial susceptibility of isolates was determined using the disc diffusion method.

**Results:**

One hundred and seven patients with bacterial meningitis were identified by cerebrospinal fluids with positive results in culture. All of patients (100%) had fever (male/female = 1.27/1). The most prevalent pathogens isolated from CSF culture were *Streptococcus pneumoniae* (34.5%), *Haemophilus influenzae *type b (23.36%), *Neisseria meningitidis* (6.54%), *Serratia *spp. (6.54%), and *Klebsiella pneumoniae* (5.6%), respectively. Moreover, the patients' blood culture had positive results in 36.44% of cases with *H. influenzae* type b (20.65%) and *S. pneumoniae *(6.54%) as the main bacteria isolated from blood. Meningitis occurred mostly in children under two years (P = 0.001). According to antimicrobial susceptibility test, a relatively high resistance was reported against some conventional cephalosporins and other antibiotics.

**Conclusions:**

*S. pneumoniae *and *H. influenzae* type b were the main pathogens of bacterial meningitis in children in the area under study. Most species had relatively high resistance to conventional antibiotics as compared to the past.

## 1. Background

Meningitis is an inflammation of the membranes (meninges) surrounding the brain and spinal cord. The disease can be caused by viral or bacterial pathogens (1, 2). Most cases of bacterial meningitis occur in childhood and its pathogens are varied in different age groups (3). *Streptococcus pneumoniae*, *Neisseria meningitidis*, and *Haemophilus influenzae *type b are among the prevalent bacterial pathogens of this disease (4-6). In recent years, two main changes have been observed in the epidemiology of acute bacterial meningitis (7). The first is a decrease in the incidence of *H. influenzae* type b and *S. pneumoniae *meningitis in countries where vaccination plan is generally performed against the two bacteria (8-10). The second is an increase in resistant strains of pneumococcus across the world (11-13). Due to the lack of vaccination against these pathogens in the area under study, acute bacterial meningitis is a fatal urgency with high rate of mortality and serious potential effects (14). Therefore, early diagnosis and appropriate antibiotic therapy are necessary to avoid further complications. However, the occurrence and etiologies of bacterial meningitis vary in different geographic regions. Moreover, effectiveness of treatment is limited due to antibiotic resistant bacterial strains (4, 15).

The epidemiology of bacterial meningitis has not been well characterized in our country so far. Furthermore, to make decision regarding children's vaccination against causative bacteria in our country, it is required to know the outbreak of the bacterial meningitis in the area. On the other hand, to make proper decision concerning the treatment of bacterial meningitis, we need to recognize antibiotic resistance patterns of prevalent bacteria in the area. This study is the first comprehensive study to assess bacterial meningitis in northwest of Iran.

## 2. Objectives

The present study was performed to investigate the etiological profile and antimicrobial resistance patterns of bacterial isolates of meningitis in children in northwest of Iran to improve evidence-based therapeutic strategies.

## 3. Patients and Methods

### 3.1. Study Population and Data Collection

In a retrospective study, of 7112 suspected cases of meningitis admitted to Tabriz Children Educational-Health Care Center (the only Children’s referral hospital in northwest of Iran) between April 2003 and March 2013, a total of 107 cerebrospinal fluid (CSF) culture had positive results for a bacterial pathogen and included in the study. Our sampling was purposive. Patients were defined as having acute bacterial meningitis if the cerebrospinal fluid (CSF) culture had positive result for at least a bacterial pathogen. Permission was obtained from patients before inclusion in the study. Some information including age, weight, sex, clinical symptoms and history of illness were recorded at the time of admission. All patients’ data were recorded and analyzed according to the considerations of university ethical committee.

### 3.2. Laboratory Methods

Standard methods were used for the analysis and culture of CSF specimens collected from all suspected patients. Immediately after receipt, each CSF specimen was centrifuged at 1500 rpm for 15 minutes. The supernatant was removed and the sediment was cultured on 5% sheep blood agar and chocolate agar plates. Gram staining was also performed. Furthermore, blood cultures were performed for each patient on admission. All isolates were identified based on their colony, morphology, culture characteristics, and biochemical reactions according to the standard microbiological procedures (16).

### 3.3. Antibiotic Susceptibility Tests

All isolates were examined for resistance to routine antimicrobial agents by standard disk diffusion method using *Staphylococcus aureus *(ATCC 25923) and *Escherichia coli* (ATCC 25922) as control strains (17). The antibiotics tested were gentamicin, amikacin, ceftazidime, ceftizoxime, cefotaxime, ceftriaxone, imipenem, ciprofloxacin, co-trimoxazole, chloramphenicol, penicillin, oxacillin, ampicillin, vancomycin, rifampicin and erythromycin (Mast Co, the UK). Isolates showing intermediate levels of susceptibility were classified as nonsusceptible.

### 3.4. Statistical Analysis

Data was analyzed using SPSS software version 13. Chi-square test was used to calculate rates and percentages. The significance level was defined as P < 0.05.

## 4. Results

During the 10-year of this study, 107 cases with confirmed acute bacterial meningitis were reported based on positive results in culture of cerebrospinal fluid. Patients included 60 males (56.07%) and 47 females (43.93%) with at least 2 days to 13 years old (mean age = 4.2 years). Clinical manifestations during hospitalization are presented in Table 1. Fever, vomiting, and meninges irritation were the most prevalent symptoms of patients. In the present study, the results of simultaneous blood culture were also examined. The most prevalent organism isolated from blood cultures was *H. influenzae* type b (20.65%). While, *S. pneumonia* (34.5%) and *H. influenzae* type b (23.36%) were the main bacteria isolated from CSF, respectively. The prevalence of other bacterial pathogens isolated from the patients' CSF and blood cultures are listed in Table 2. According to our results, 70.09% of patients were under 2 years, which 89.18% of them had pneumococcal meningitis (P = 0.001).

According to antimicrobial susceptibility testing, most species were identified to have relatively high resistance against some conventional antibiotics including the third-generation cephalosporins. Table 3 shows the antimicrobial resistance pattern of pathogens isolated from CSF culture.

**Table 1.** Clinical Manifestations of Children With Acute Bacterial Meningitis

Clinical Symptoms	Number of Patients	Occurrence (%)
**Fever**	107	100
**Vomiting**	80	74.76
**Meninges irritation**	75	70.09
**Seizure**	43	46.01
**Headache**	17	15.88
**Lethargy**	41	37.38
**Stupor**	11	10.28
**Coma**	7	6.54

**Table 2.** Isolated Organisms From CSF and Blood Cultures of Patients With Acute Bacterial Meningitis

Microorganisms	CSF, No. (%)	Blood, No. (%)
***Streptococcus pneumoniae***	37 (34.5)	7 (6.54)
***Haemophilus influenzae***	25 (23.36)	22 (20.65)
***Neisseria meningitidis***	7 (6.54)	3 (2.8)
***Serratia*** **spp.**	7 (6.54)	1 (0.93)
***Klebsiella pneumoniae***	6 (5.6)	2 (1.86)
***Pseudomonas aeruginosa***	4 (3.73)	1 (0.93)
***Escherichia coli***	4 (3.73)	-
***Staphylococcus aureus***	4 (3.73)	1 (0.93)
**Viridans** *** Streptococci***	4 (3.73)	1 (0.93)
**Coagulase-Negative Staphylococci (CoNS)**	3 (2.8)	1 (0.93)
***Enterococcus*** ** spp.**	3 (2.8)	-
***Acinetobacter*** ** spp.**	2 (1.86)	-
***Salmonella *** **serogroup D**	1 (0.93)	-

**Table 3.** Antimicrobial Resistance (%) of Bacterial Pathogens Isolated From CSF Culture

Antibiotics	*S. pneumoniae*	*H. influenzae*	*N. meningitidis*	*K. pneumoniae*	*E. coli*	*Serratia *spp.	*P. aeruginosa*	*S. aureus*	CoNS	*Viridans Streptococci.*	*Enterococcus *spp.	*Acinetobacter *spp.	*Salmonella*
**Ciprofloxacin**	8	4	0	0	25	15	60	50	-	75	66.66	50	0
**Gentamicin**	29.72	16	0	87.5	50	57.14	40	50	75	100	100	100	0
**Amikacin**	35.13	24	0	100	50	100	20	25	25	100	100	50	0
**Vancomycin**	0	-	-	-	-	-	-	0	0	25	33.33	-	-
**Rifampicin**	2.7	28	-	-	-	-	-	25	25	50	33.33	-	-
**Erythromycin**	16.21	-	-	-	-	-	-	50	75	75	100	-	-
**Imipenem**	-	0	-	12.5	25	20	20	-	-	-	-	50	-
**Co-trimoxazole**	32.43	20	0	62.5	25	14.28	100	25	-	75	33.33	100	100
**Chloramphenicol**	5.4	0	0	12.5	50	57.14	60	75	-	50	33.33	100	0
**Ceftriaxone**	10.81	20	0	62.5	100	85.71	60	75	75	25	100	100	0
**Cefotaxime**	8.1	28	0	87.5	75	85.71	60	50	75	25	100	100	0
**Ceftizoxime**	13.51	20	0	62.5	75	85.71	60	50	75	50	100	100	0
**Penicillin**	37.83	-	30	-	-	-	-	100	100	100	100	-	-
**Oxacillin**	48.64	-	-	-	-	-	-	50	75	75	100	-	-
**Ampicillin**	-	75	-	-	-	-	-	-	-	-	33.33	-	100
**Ceftazidime**	8.1	20	-	100	100	85.71	25	-	-	-	100	100	0

## 5. Discussions

Bacterial meningitis is still one of the main health problems in children and newborns (2, 3). Etiological pathogens of meningitis are relatively diverse (8). Most researchers have introduced *S. pneumoniae*, *H. influenzae* type b, and *N. meningitidis* as the main pathogens of the bacterial meningitis especially in childhood (6). According to previous studies, prevalence of these pathogens can be different based on time, geographical area, and patient's age (3, 18). In an extensive study regarding the etiology of acute bacterial meningitis in some of the third world countries, Laxer and Marks reported *S. pneumoniae *as the most prevalent pathogen in children (11). We found that the most prevalent organisms isolated from CSF were *S. pneumoniae*, *H. influenzae* type b, *N. meningitidis*, and *Serratia *spp., respectively. This finding is in close accordance with studies performed in countries where meningitis vaccine is not administered (11, 19). Meanwhile, according to the results of previous studies conducted in other areas of Iran, *S. pneumoniae *and *H. influenzae* type b were the main bacteria isolated from CSF, respectively. Nevertheless, a report in Turkey showed that *N. meningitidis* serogroup W135 was the dominant organism isolated from children with bacterial meningitis (20). Moreover, recent studies indicated that outbreak level of *S. pneumoniae*, *H. influenzae* type b, and *N. meningitidis* significantly has reduced in some developed countries due to the administration of vaccines (9, 10, 13, 21). Although, *Listeria monocytogenes* and *Streptococcus agalactiae* are discussed as the important pathogens of bacterial meningitis in other areas except Iran (22-24); these bacteria were not isolated from CSF cultures in our study. It is not clearly known, whether these bacteria are not truly prevalent in our country or this is due to the lack of standard techniques required for their isolation. Furthermore, previous prescription of antibiotics leads to false negative results in culture and a decrease in the sensitivity of culture techniques, which may also affect our results. In this study, we investigated only culture-positive bacterial meningitis in children with bacterial meningitis symptoms and those with negative results in cultures were excluded. As shows in Table 3, the antimicrobial susceptibility testing of the isolates demonstrated that *S. pneumoniae *had high resistance against penicillin. Moreover, these isolates had intermediate resistance against the third generation cephalosporins. These results represent an alarming and disturbing rate of resistant pneumococcus species in northwest Iran. Yet, resistance against vancomycin was not reported in our study, except one isolate of viridans *Streptococcus* as well as one *Enterococcus* species. In addition, the results indicated that the resistance of *H. influenzae* isolates against the third generation cephalosporins was considerable and about one third of the isolated strains were resistant against rifampicin, but susceptible to imipenem. Regarding *N. meningitidis*, no resistance was reported against cephalosporins and there was only resistance against penicillin. Other Gram-negative bacteria had relatively high resistance against aminoglycosides and β-lactams. Table 3 shows the frequency distribution of antimicrobial resistance of the main isolated bacteria against used antibiotics.

Blood culture is valuable to detect the causative organism and establish susceptibility patterns if CSF cultures have negative results or be unavailable. However, blood culture positivity differs for each causative organism (24). In present study, blood culture was effective in 15% of samples, where the most prevalent organism isolated from blood culture of patients was *Haemophilus influenzae *type b.

Finally, our study revealed that emergence of resistant *S. pneumoniae *and *H. influenzae* strains needs continuous monitoring of antibiotic susceptibility patterns of clinical isolates for the appropriate selection of empirical therapy. Moreover, our results indicated that vaccination against *S. pneumoniae *and *H. influenzae* type b seems to be necessary.
